# The Prevalence and Associated Factors of Hypertension among Adults in Southern Ethiopia

**DOI:** 10.1155/2020/8020129

**Published:** 2020-04-11

**Authors:** Belachew Kebede, Gistane Ayele, Desta Haftu, Gebrekiros Gebremichael

**Affiliations:** ^1^Public Health, John Snow Inc., Ethiopia; ^2^School of Public Health, Arba Minch University, Ethiopia; ^3^School of Public Health, Mekelle University, Ethiopia

## Abstract

**Background:**

Hypertension is a growing public health problem in many developing countries including Ethiopia. Determining the prevalence of hypertension and identifying the associated factors is crucial.

**Objective:**

To assess the prevalence of hypertension and associated factors, among adult population of Arba Minch town, Gamo Zone, Southern Nations, Nationalities and Peoples Region, Ethiopia.

**Methods:**

A cross-sectional study design was conducted from December 1 to 30, 2017 among adults. Study participants were selected using a multistage systematic sampling method. Data were collected by face-to-face interview after getting written informed consent by using a structured questionnaire. Additionally, weight, height, and blood pressure of participants were measured following standard procedures. Data were entered into a computer using EPI INFO 7 and exported into SPSS version 20 for analysis. Bivariate and multivariable analyses were performed to explore the association between hypertension and associated factors. Multivariable logistic regressions were fitted to control the effect of confounders.

**Results:**

A total of 784 study participants were included in this study. The overall prevalence of hypertension in Arba Minch Town was 35.2%, (95% CI: 32.4%, 38.4%). Nearly 90% of hypertensive patients were screened for the first time. Age ≥55 years [AOR = 7.74; 95% CI: 2.19, 27.23], income level which is greater than 2501 Ethiopian Birr [AOR = 9.5; 95% CI: 4.5, 20.20], working hour less than seven hours per day [AOR = 12.5; 95% CI: 4.3, 36.1], and chewing “khat” [AOR = 11.06: 95% CI: 4.3, 27.7] were the independently associated factors with hypertension.

**Conclusion:**

The prevalence of hypertension is found to be high. Increasing awareness on control use of “khat,” increasing physical activity, and strengthening community-based periodic screening programs of high-risk populations are recommended.

## 1. Introduction

Noncommunicable diseases (NCDs) are a major cause of morbidity and mortality globally accounting for about three-fourths of all deaths worldwide [1]. Hypertension is one of the main public health challenges because of its high frequency and associated risks of cardiovascular and kidney diseases such as myocardial infarctions, strokes, and renal failures [2, 3]. Hypertension is the top leading cause of the global burden of disease [4]. The African region did not differ from the global epidemic of NCDs, and, in fact, suffered from the double burden of diseases (communicable and noncommunicable diseases) [5]. The World Health Organization (WHO) predicted deaths from NCDs would increase globally by 17% over the next ten years where the greatest increase will be in the African region (by 27% or 28 million deaths from NCDs) [6].

Little is known about the magnitude and factors of hypertension in Ethiopia; although, recent evidences indicate that hypertension and raised blood pressure are increasing partly because of the increase in risk factors including smoking, obesity, use of alcohol, and lack of exercise [7].

A systematic meta-analysis from Ethiopia showed about 19.6% prevalence of hypertension with a higher proportion in urban than rural [8]. Similarly, a 15.8% prevalence of raised blood pressure was reported from the national NCDs STEPS survey in 2015 [9].

An up-to-date and comprehensive assessment of the evidence concerning hypertension in Ethiopia is lacking. On the other hand, urbanization is expanding, lifestyles are changing, the literacy rate is low, and people are still living in poverty. Thus, this study assessed the prevalence and associated factors of hypertension among the adult population of Arba Minch town.

## 2. Methods

### 2.1. Study Settings

A community-based cross-sectional study was conducted among adult population living in Arba Minch town from December 1 to 30, 2017. The study was done at Arba Minch Town, which is zonal capital city, and it is 505 km from Addis Ababa and 273 km south of the regional capital Hawassa. Based on figures obtained from 2007 National Population and Housing Census of Ethiopia, the adjusted population projection of the total population of the Arba Minch town was 110,104, of whom 54,833 (49.8%) were males and 55,271 (50.2%) were females. Arba Minch town is administratively divided into four subcities, namely, Secha, Sikela, Nechsar, and Abaya. The town had two health centers, 11 kebeles, and 38 urban health extension workers. The town has a total of 57,397 (53.13%) adult population, which means ages 15 years and above [10].

### 2.2. Population

The source population were all adult persons living in Arba Minch town, and the study population were all selected adult populations who live in the town during the study period. All adult persons whose age is 18-70 years and lived at least 6 months in the town were included. Those who were disabled individuals, pregnant women, known hypertensive patients (self-declared), and persons who were severely diseased were excluded from the study.

### 2.3. Sample Size and Sampling Procedures

The required sample size for this particular study was determined by the Epi Info version 7 software using a single-population proportion formula *n* = (*z*2^∗^ P (1 − P)/*d*2), where “*n*” is the sample size, “*z*” is the standard normal score set at 1.96, “*d*” is the desired degree of accuracy, and “*p*” is the estimated proportion of the target population. By taking, *p* = 25.1% [11], *z* = 1.96 and *d* = 4.5%. Based on these assumptions, the final sample size with the design effect of 2 and 10% for nonresponse rate was 784.

Arba Minch town has 11 administrative units or kebeles. Multistage systematic sampling was used, and at first stage, five of the eleven kebeles (45.5%) were selected by lottery method to get representative samples of the population. The second stage was used to select study households by using systematic sampling method, and the sample was proportionally distributed to each selected kebeles. Total number of households was obtained from the town administrative office.

Then, after determining the number of individuals to be studied in each kebele, the sample size in each kebele was divided by the number of households in the kebele to determine the proportion of individuals to be studied in each selected kebele. After getting the sampling fraction in the selected kebeles or administrative units, systematic sampling was used to select the households and a lottery method was used to select the first household. Then, every 3rd unit of households were visited to get the required number of study participants in all selected kebeles of the city. If more than one eligible adult was present in the selected household, one household member was selected randomly using lottery method.

### 2.4. Variables of the Study

#### 2.4.1. Dependent Variables

Presence or absence of hypertension.

*(1) Independent Variables*.

- **Socio-demographic variables**: Age, gender, marital status, family history of hypertension, height, weight, body mass index, feeding practice, and sedentary lifestyle (physical inactivity).
- **Socio-economic variables**: Occupation, monthly income, highest education attained, history of smoking, and alcohol consumption.

*(2) Definition of Terms*. **Hypertension**: the average of casual systolic Blood Pressure readings ≥140 mmHg and/or diastolic pressure readings ≥90 mmHg [7, 12].

**Sedentary lifestyle** (physical inactivity): is measured as a response of being always or usually engaged in light/leisure activities for most days of the week or a response of sometimes/never engagement in moderate to intense physical activity outside work for most days of the week that would add up to at least three hours per week of moderate to intense (vigorous) physical activity [13].

**Sufficient Fruit and Vegetable Intake**: Intake of seven-day history was use and 4-7 days use of fruit and vegetables in a day was regarded as sufficient [12].

**Khat**: stimulant leaves of a shrub that have a stimulating and euphoric effect when chewed [14, 15].

### 2.5. Data Collection Procedures

The data were collected using a structured questionnaire through interview and physical measurements. The questionnaire was adapted from the “WHO STEP wise approach to chronic disease risk factor surveillance (STEPS)” [16].

A digital measuring instrument was used to measure the weight of adult individuals to the nearest 100 grams. Weight measuring scales were checked and adjusted to zero levels between each measurement. Height was measured with stadiometer following the standard steps. Blood pressure was measured twice in a sitting position (using a standard sphygmomanometer blood pressure cuff with an appropriate size to cover two-thirds of the upper arm) after the participant rested for at least 5 minutes, with no smoking or caffeine allowed for 30 minutes before measurement. The second measurement was taken 5 minutes after the first measurement. Finally, the mean of the two measurements was taken to determine hypertension. Eight clinical nurses were selected as data collectors, and two experienced health professionals were recruited as supervisors for the fieldwork.

### 2.6. Data Quality Management

Quality of data were assured by using a structured questionnaire which was translated into Amharic language, back-translated into English, and pretested before the actual survey. A pretest was done among 5% of the sample size among individuals in kebeles that were not included in the study. Data collectors and supervisors were trained for three days on procedures of measuring blood pressure, heart rate, weight, and height of the participants and were also made familiar with the questionnaire.

### 2.7. Data Processing and Analysis

After the questionnaires were checked for errors and coded, data were entered and cleaned using EPI info version 7 software. Then, it was exported into SPSS version 20 software package for further cleaning and analysis. Data were cleaned by doing simple frequency and cross-tabulation between each independent and dependent variable. Body mass index (BMI) was calculated dividing the weight in kilograms of the participants by the squares of their height in meters. Then, it was categorized as underweight (less than 18.5), normal (18.5-24.9), overweight (25-29.9), and obese (30 and above). Cross-tabulation and bivariate logistic regression were used to explore the relation between hypertension and the different independent variables using crude odds ratio (COR) with 95% CI. Finally, to determine the independent factors associated with hypertension, multivariable logistic regression model was done. Model fitness was checked using Hosmer-Lemeshow test which was 0.23. Variables with *p* < 0.05 in the multivariable analysis were considered significant and presented by adjusted odds ratio (AOR) with 95% CI.

## 3. Results

### 3.1. Socio-Demographic Characteristics

A total of 784 participants of ages 18 and above years participated in the study yielding in 100% response rate. Of the total respondents, 344 (43.9%) were males and 440 (56.1%) were females. Nearly half of the study participants (41.5%) were in the age group of 45-54 years followed by 35-44 years (30.1%). Majority of the participants can read and write (89%), were married (74.4%), and had a family history of chronic health diseases (71%). The median monthly income of the respondents was 4500ETB (Table 1).

### 3.2. Prevalence of Hypertension

The prevalence of adult hypertension was 35.2% (95% CI: 32.4%, 38.4%). The prevalence of hypertension was 54.7% among females and 44.9% among males. Among the hypertensive cases, around ninety percent 251 (90.9%) were newly screened for the first time but the twenty-five participants (9%) had been screened for hypertension before.

### 3.3. Behavioral Characteristics of Study Participants

Overall, 201 (25.6%) respondents reported that they have ever smoked cigarettes in their lifetime. Eighty-three (10.6%) respondents have ever chewed “khat.” Among the study participants, 510 (65.1%) reported that they ever took alcoholic drinks in their lifetime. Daily drinkers were, however, found in only 22 (8.0%) of all ever drinkers who constitute 34.9% of the total study participants. Out of the total study respondents, 208 (26.5%) reported that they regularly have moderate to intense physical activities some times in a week, but the rest 576 (73.5%) did not.

### 3.4. Biological Characteristics of Study Participants

The body mass index (BMI) of the respondents ranged from 14.6 to 33.8 kg/m^2^, with a mean of 24.5 ± 2.9 kg m^2^. Overall, 330 (42.0%) of participants had BMI < 25.0 kg/m^2^, and 454 (57.9%) of respondents had BMI ≥ 25.0 kg/m^2^. Three hundred sixteen (40.3%) participants were overweight and 14 (1.8%) were obese. The mean ± SD systolic blood pressure of the study population was 137.9 ± 16.5 mmHg, while the diastolic mean ± SD was 86.2 ± 10.9 mmHg.

### 3.5. Factors Associated with Hypertension

In the bivariate analysis of the socio-demographic variables with hypertension, age was found to have a statistically significant association with the more odds of hypertension among the age group 45-54, (COR = 4.9; 95% CI: 2.1, 11.2), and those ≥55 years, (COR = 8.95; 95% CI: 3.6, 22.6), compared to the younger age group (18-24 years).

An increase in the odds of hypertension with increasing income level was noted in the bivariate analysis, and it was statistically significant. Those who earned between 1,001 and 2,500 ETB, (COR = 3.8; 95% CI: 2.09, 6.83), and >2501ETB, (COR = 5.9; 95% CI: 3.6, 9.7), were more likely having hypertension compared to those earning less than one thousand ETB. Sex and marital status did not show any statistically significant association with hypertension in the study population.

Among the behavioral characteristics, being obese was found to be positively associated with the odds of hypertension. Those who had body mass index greater or equal to 30 kg/m2 more likely to have hypertension (COR = 10.0; 95% CI: 2.3, 43.2) than those with a BMI lower than 30 kg/m^2^.

Similarly, a positive association was found for “khat” chewers. Using “khat” regularly had more chance to develop hypertension than nonchewers, (COR = 23.0; 95% CI: 11.0, 49.2), and also working hour also has an association; those working less than seven hours daily are less likely to develop hypertension (COR = 22.9; 95% CI: 9.2, 57.1).

In the same way using fruits and vegetables have an association with hypertension, those peoples using fruits and vegetables 4-7 days per week were less likely to develop hypertension when compared to not consuming totally (COR = 0.024; 95% CI: 0.003, 0.22). Similarly, using salt always in food is associated with more likelihood to develop hypertension when compared to not using salt all (COR = 11.5; 95% CI: 4.1, 31.8). Other behavioral variables such as smoking, alcohol drinking, and transportation method used did not show any significant associations with hypertension in the study participants (Table 2).

In the multivariable logistic regression analysis, age group 45-54 and ≥55 years, proved to have an independently significant association with hypertension among the study participants. Persons in the age group 45-54 and ≥55 years were found to be 6 and 7.7 times at more odds of having hypertension than 18-24 years of age (AOR = 6.5; 95% CI: 2.1,20.7 and AOR = 7.74, 95% CI: 2.19, 27.23), respectively.

Income level of participants also showed an independent association with hypertension. Respondents with income level 1,001-2,500 ETB and ≥2,501 ETB were found to be almost five and nine times at more likelihood of having hypertension than those families whose income level was ≤1,000ETB (AOR = 5.1; 95% CI: 2.19, 12.14 and AOR = 9.5; 95% CI: 4.5, 20.2).

Respondents who work less than seven hours per day were 10.29 times more likely to develop hypertension when compared to respondents working more than eleven hours per day (AOR = 10.29; 95% CI: 3.29, 32.14).

Respondents who had ever chew “khat” were found to be more likely of having hypertension (AOR = 11; 95% CI: 4.3, 27.7) than those who do not chew “khat.” Although usage of salt in food, body mass index, and use of fruit and vegetables showed significant association with hypertension in the bivariate analysis, the variables were not statistically significant in the final logistic regression analysis.

## 4. Discussion

The prevalence of hypertension in this study was 35.2%. The finding is slightly comparable to a community-based study among police officers in Bengal, India 32% [17]. The prevalence of hypertension in this study is higher than the studies in Gonder with the prevalence of 28.3% (26), Addis Ababa 31.5% [7], among a ministry of civil servants 27.3% [18], Durame 22.4%, Bahir Dar city 25.1% and 27.3% [2, 19, 20], Ethiopia and Hawwasa 19.6% [8, 21], Jigjiga 28.3%, and Tigray 16% [22, 23]. This discrepancy may be explained by the study methodology and setting difference. Secondly, the time of study may make such a difference since the prevalence of hypertension is increasing.

The prevalence of hypertension in this study was lower than that reported in Ghana ranged from 19 to 48% between studies [12], and those reported from population-based studies is 55.9% [24, 25]. This discrepancy may be due to age difference as 18-70 years were used in this study and others use 30 years and above. On top of this, genetic difference may probably affect the prevalence of hypertension.

In this study age group, 45-54 and ≥55 years were found to have a significantly associated with hypertension even after adjusting for confounders, which has been confirmed in previous studies [23, 25]. This may be because of the biological effect of increased arterial resistance due to arterial thickening as one gets older. High-income level of families, less working hour per day, and regular “khat” chewing were significantly associated with hypertension [19]. This study further strengthens the previous reports in this country.

On the other hand, being literate, cigarette smoking, regular use of alcohol, gender, and positive family history of hypertension were not significantly associated with hypertension in this study.

No significant statistical associations were found in this study between hypertension and using salty foods, sedentary lifestyles, use of fruits and vegetables, and religion. This was not in line with other studies which presented paradoxical results [14, 25]. Such differences maybe due to disparities in methodologies and due to the fact that majority of the participants in this study were females (56%), who are less smokers and alcohol users compared to males. This study adds to the evidence that the prevalence of hypertension in Ethiopia is on the rise.

This study was not free of limitations. It involves only physical and behavioral measurements but did not used the biochemical tests due to cost and time constraints. Social desirability bias might have been introduced in “khat” chewing, smoking, and alcohol drinking practices especially for women. The blood pressure measurements were taken in a single day. This study also did not measure the effect of “khat” chewing frequency on hypertension. Another limitation is we used systematic sampling which may not guarantee randomness, and those households who were not owners and/or rented small rooms in the compound were not included.

## 5. Conclusion

This study has found a higher prevalence of hypertension than previously reported in urban populations in the country. Advancing age, regular “khat” chewing, increased income, and working hour in relation to physical exercise were the identified associated factors with hypertension.

## Figures and Tables

**Table 1:** Sociodemographic characteristics of the study participants at Arbaminch town, Gamo, Ethiopia, 2018.

Variables		Number (*n* = 784)	Percent (%)
Sex	Male	344	43.9
	Female	440	56.1

Age group	18-24	54	6.9
	25-34	98	12.5
	35-44	236	30.1
	45-54	326	41.6
	≥55	70	8.9

Educational status	Can read and write	698	89
	Cannot read and write	86	11

Marital status	Married	583	74.4
	Single	111	14.2
	Divorced/widowed	67	8.5

Income (ETB)	≤1,000 ETB	171	21.8
	1,001-2,500 ETB	129	16.5
	≥2,501 ETB	484	61.7
Family history of chronic heart diseases	No	226	29
	Yes	558	71.1

**Table 2:** Factors associated with hypertension of the study participants in Arba Minch town, Gamo, Ethiopia, 2018.

Variables		Hypertension		COR (95% CI)	AOR (95% CI)
		Yes	No		
Age	18-24	7	47	1	1
	25-34	15	83	1.21 (0.5, 3.2)	1.5 (0.5, 5.4)
	35-44	76	160	3.19 (1.4, 7.2)	4.49 (1.4, 14.5)
	45-54	138	188	4.92 (2.2, 11.2)^∗^	6.5 (2.1, 20.7)^∗^
	≥55	40	30	8.95 (3.6, 22.6)^∗^	7.74 (2.2, 27.2)^∗^

Income	≤1,000 ETB	20	151	1	1
	1,001-2,500 ETB	43	86	3.8 (2.1, 6.8)^∗^	5.16 (2.2, 12.1)^∗^
	≥2,501	213	271	5.9 (3.6, 9.8)^∗^	9.5 (4.5, 20.2)^∗^

“Khat” users	No	201	500	1	1
	Yes	75	8	23.(11, 49.2)^∗^	11.06 (4.3, 27.7)^∗^

Working hour	≤7 hours per day	70	7	22.9 (9.2, 57.1)^∗^	12.5 (4.3, 36.1)^∗^
	8-10 hours per day	182	405	0.94 (0.6, 1.6)	0.5 (0.3, 0.9)
	≥11 hours per day	24	96	1	1

Use of fruits and vegetables	Not at all	6	1	1	1
	1-3 days per week	261	444	0.1 (0.012, 0.8)	34 (1.9, 585.3)
	4-7 days per week	9	63	0.02 (0.01, 0.2)^∗^	4 (1.1, 14.2)

Use of salt	Not at all	4	73	1	1
	Always	237	376	11.5(4.1, 31.9)^∗^	3.1 (0.9, 10.2)
	Some times	35	59	10.8 (3.6, 32.2)^∗^	2.9 (0.8, 10.3)

BMI	≤17.99 under weight	6	24	1	1
	18-24.99 normal	130	294	1.7 (0.7, 4.4)	1.0 (0.3, 3.3)
	25-29.99 over weight	130	186	2.8 (1.1, 7.0)	0.8 (0.2, 2.8)
	≥30 obese	10	4	10 (2.3, 43.3)^∗^	3.n (0.4, 23.0)

^∗^ denotes statistical significance (*p* value < 0.05).

## Data Availability

The datasets used and/or analyzed during the current study are available from the corresponding author on reasonable request.

## Abbreviations

BMI: Body mass index
NCD: Noncommunicable disease
WHO: World Health Organization.

## Additional Points

*Paper Context*. Nowadays, Ethiopia is facing a double burden of both the communicable and noncommunicable diseases. Hypertension, as one of the major killer noncommunicable diseases, little is known about its prevalence and contributing factors in the adult population. An up-to-date and comprehensive assessment of the evidence concerning hypertension in Ethiopia is lacking. A prevalence of hypertension is found in the adults which urges for regular screening and comprehensive health education and promotion activities on regular physical exercise.
